# A systematic review of influenza A virus prevalence and transmission dynamics in backyard swine populations globally

**DOI:** 10.1186/s40813-022-00251-4

**Published:** 2022-03-14

**Authors:** Ravendra P. Chauhan, Michelle L. Gordon

**Affiliations:** School of Laboratory Medicine and Medical Sciences, Nelson R. Mandela School of Medicine, College of Health Sciences, University of KwaZulu-Natal, 719 Umbilo Road, Durban, 4001 South Africa

**Keywords:** Avian influenza, Backyard swine farming, Biosecurity, Influenza A virus, IAV outbreak, IAV pandemic, Interspecies IAV transmission

## Abstract

**Background:**

Backyard swine farming is critical to generating subsistence and food security in rural and peri-urban households in several developing countries. The objective of this systematic review was to analyze the molecular and serological prevalence of influenza A virus (IAV) in backyard swine populations globally.

**Results:**

We identified 34 full-text research articles in NCBI-PubMed and Google Scholar databases that have reported IAV sero- and/or virological prevalence in backyard swine up to 11 July 2021. The highest number of studies were reported from Asia (n = 11) followed by North America (n = 10), South America (n = 6), Africa (n = 6), and Europe (n = 1). While the maximum number of studies (44.12%) reported human-to-swine transmission of IAV, swine-to-human (5.88%), poultry-to-swine (5.88%), and wild birds-to-swine (2.94%) transmissions were also reported. An overall higher IAV seroprevalence (18.28%) in backyard swine was detected compared to the virological prevalence (1.32%). The human-origin pandemic A(H1N1)pdm09 virus clade 1A.3.3.2 was the more frequently detected IAV subtype in virological studies (27.27%) than serological studies (18.92%). In addition, the avian-origin highly pathogenic H5N1 and H5N8 viruses were also detected, which further substantiated the evidence of avian–swine interactions in the backyards.

**Conclusion:**

Human–swine and avian–swine interactions in backyards may transmit IAV between species. Monitoring the circulation and evolution of IAV in backyard swine would help stakeholders make informed decisions to ensure sustainable backyard swine farming and public safety.

## Background

**Fig. 1:**
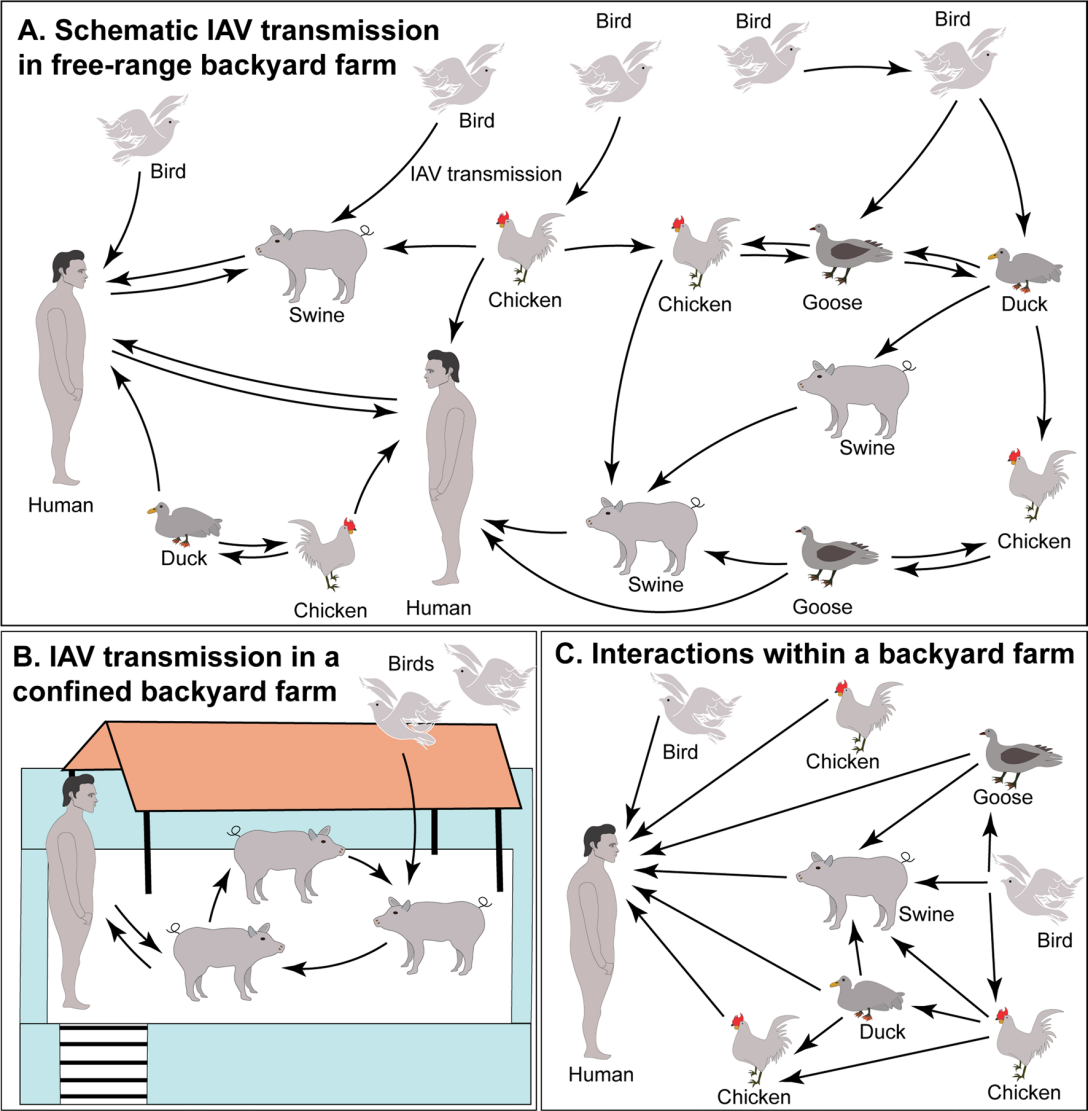
A schematic representation of IAV transmission within the backyard farms. **a** Birds may disseminate IAV strain(s) to the poultry and/or swine. A high probability remains for the zoonotic and reverse-zoonotic transmission of IAV between swine and household members, which may also trigger human-to-human transmission. **b** A schematic representation of IAV transmission in backyard swine kept within the pens. Birds may disseminate IAV to the swine confined in the pens, resulting in swine-to-swine transmission. Occupational exposure may facilitate zoonotic or reverse zoonotic IAV transmission between swine and household members. The IAV transmission may occur in both production types through contaminated feed, water, or bird faeces. **c** Humans, swine, chickens, ducks, geese, and birds may frequently interact within the backyard which increases the risk of IAV transmission among them

Swine farming is the largest meat-producing industry, with gross production of more than one-third of all the meats consumed globally [1, 2]. While pork is produced mainly by large-scale commercial farms to meet the demand, many small-scale backyard farms co-exist, primarily for subsistence and food security, within the rural or peri-urban households in developing countries [2]. A backyard farm is a household unit in rural, agrarian, or peri-urban communities that rears one or more animal species, including swine (*Sus scrofa domesticus*), chicken, ducks, turkey, and cattle, raised for either household consumption or supplying within the local community for subsistence. One prominent challenge backyard swine farming faces is the lack of suitable biosecurity, which may facilitate the dissemination of zoonotic pathogens, including influenza A virus (IAV), endangering backyard farming and public health. Negligence of biosecurity at the backyard farms may provide a suitable environment for disseminating IAV within the backyard animals, resulting in IAV disease outbreaks causing economic losses to the swine growers [3–10]. Several reports in the recent past have documented highly and low pathogenic avian influenza viruses in migratory and other birds [11–15] that can disseminate the IAV strains between the countries and continents [16]. Interactions of wild birds with domestic poultry and swine may transmit the IAV in the backyards. Additionally, the probability of zoonotic and reverse zoonotic transmission of IAV between swine and occupationally exposed household members threatens public health. A schematic representation of IAV transmission in backyards is illustrated in Fig. 1.

For the first time, we reported the serological and molecular prevalence and the transmission dynamics of IAV exclusively in backyard swine populations globally. This study describes the existing IAV circulation dynamics in backyard swine farms, which is essential to assess the current threat regarding the IAV disease outbreak. This study would also assist stakeholders in making an informed decision regarding minimizing the risk of IAV disease dissemination in backyard farms to ensure sustainable backyard swine farming.

## Methods

### Search criteria

**Fig. 2:**
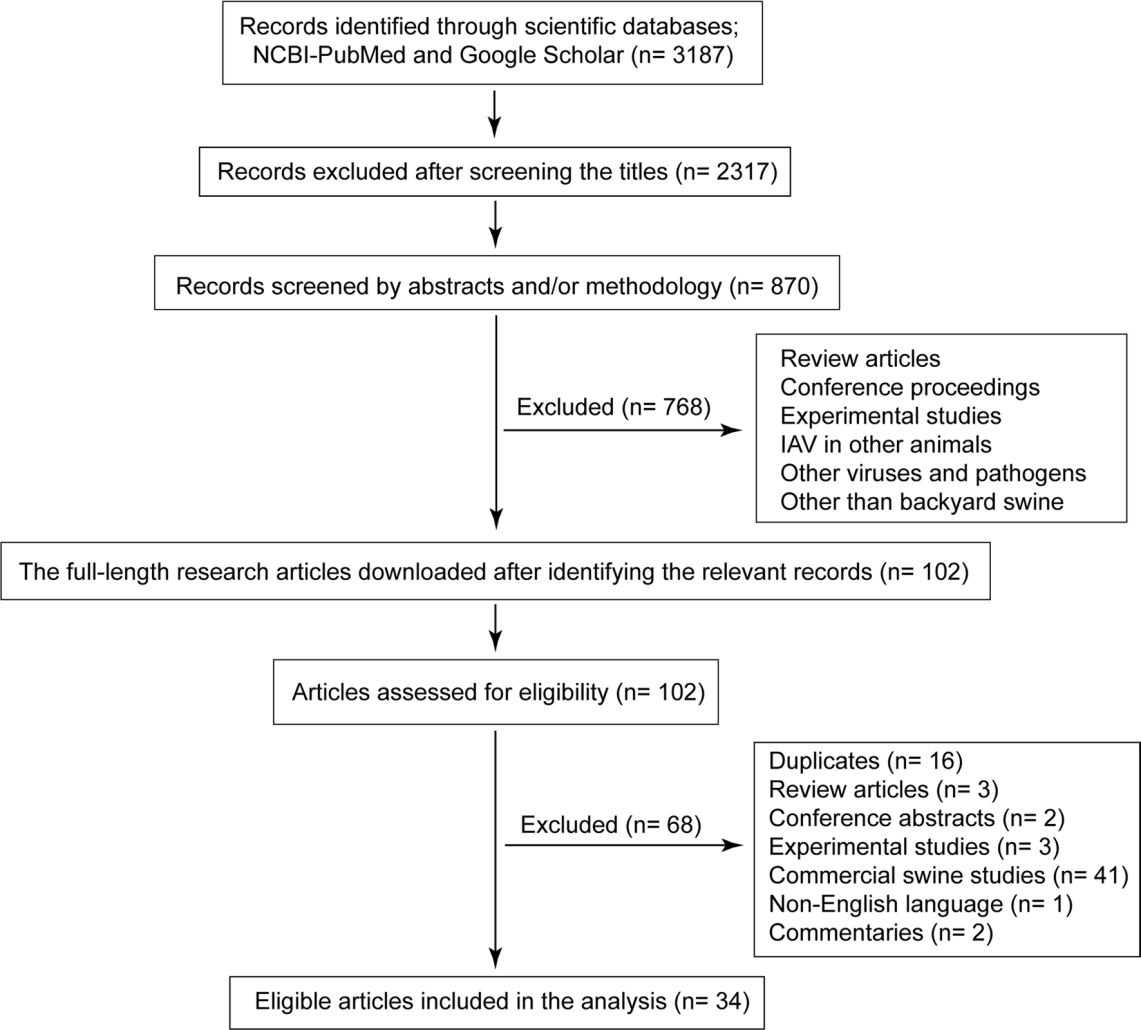
PRISMA chart illustrating the search strategy for identifying the relevant full-text research articles available in NCBI-PubMed and Google Scholar databases up to 11 July 2021 for inclusion in the study. Full-text original research articles which investigated molecular and/or serological prevalence of IAV exclusively in backyard swine populations were included in systematic review

Our objective was to analyze the transmission of IAV to the swine populations raised in the backyards, which may occur through swine–swine, poultry–swine, human–swine, and wild birds–swine interactions. A comprehensive search of NCBI-PubMed and Google Scholar databases was conducted up to 11 July 2021 for identifying the available research articles reporting IAV disease in backyard swine populations globally. The search terms including “Influenza A virus in backyard swine,” “Influenza A virus in backyard pigs,” “Influenza A virus in backyard production systems,” “Influenza A virus in rural pigs,” “Influenza A virus in household pigs,” and “Swine backyard production systems” were entered in Google Scholar and NCBI-PubMed databases one by one for identifying relevant full-text research articles. The research articles from the database search investigating IAV serological and/or molecular prevalence in backyard animals, including swine, poultry, and cattle, were segregated into groups for analysis based on the interaction patterns. An overview of Preferred Reporting Items for systematic reviews and meta-analysis (PRISMA) flowchart [17] used to screen the relevant articles is depicted in Fig. 2.

### Inclusion and exclusion criteria

The full-text original research articles which investigated molecular (virological) and/or serological prevalence of IAV exclusively in the backyard or household swine populations were included in this systematic review. The analysis did not include research articles that reported IAV sero- and virological prevalence in commercial, feral, exhibition swine, and wild boars. Conference abstracts, review articles, editorials, letters, commentaries, and opinions were not included in the analysis. We excluded the conference abstracts (n = 2) from the analysis because they did not provide sufficient information on the methodology as well as the sample size in one of the studies. Only English-language articles were included in the analysis. The relevant records were thoroughly screened through the titles, abstracts, and/or methodology, emphasizing the swine holding types for determining their relevance for inclusion in the study. The references of the identified relevant research articles were also screened to find other eligible research articles to be included in the analysis. All the relevant full-text research articles were downloaded for a detailed analysis.

## Results

**Fig. 3:**
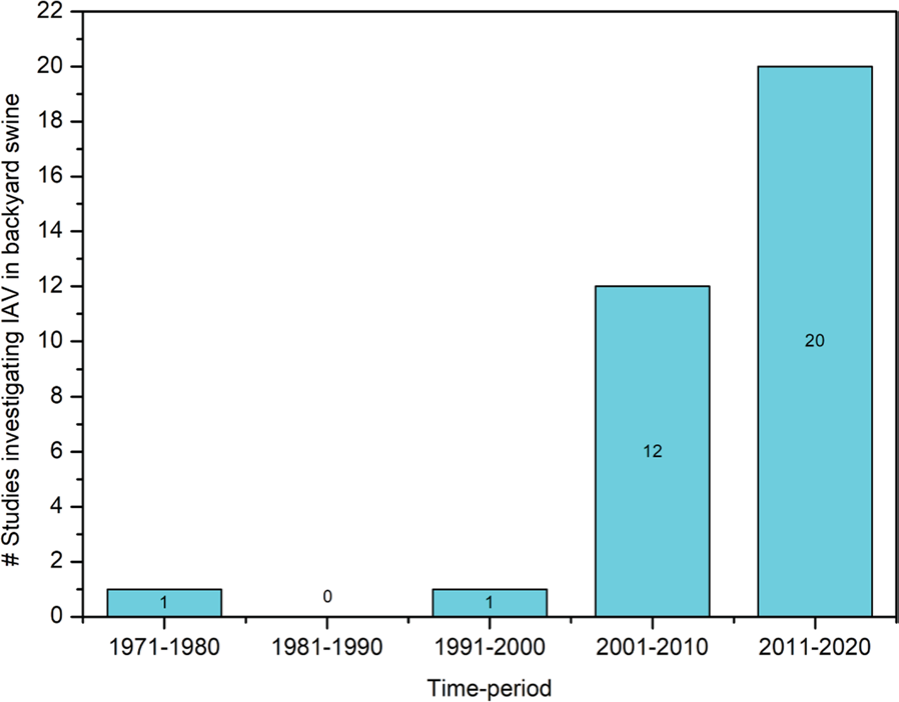
The trend of IAV investigations in backyard swine populations globally

The first study that documented H1N1 virus antibodies in backyard swine sera was reported from a family backyard farm in Wisconsin, USA, in 1977 [18]. After that, 33 other studies have been conducted for detecting IAV sero- and/or virological prevalence in backyard swine populations in 26 countries. Interestingly, most of these studies (n = 32) were conducted in the last two decades (Fig. 3), suggesting that the IAV surveillance in backyard swine populations attracted significant attention only during the recent decades.

**Fig. 4:**
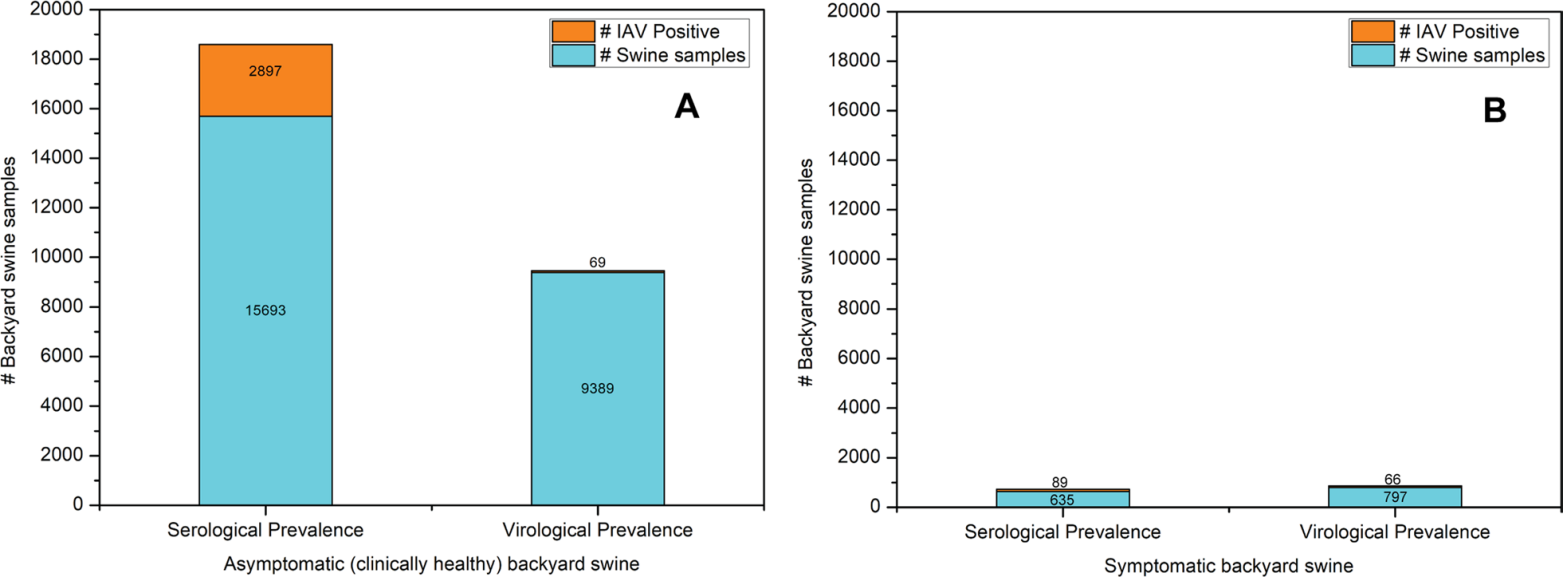
Serological and virological prevalence of IAV in **a** asymptomatic (clinically healthy) and **b** symptomatic (sick) backyard swine populations globally

The studies included active and passive IAV surveillance in backyard swine from various countries, including clinically healthy and symptomatic swine. The clinical signs of IAV disease symptoms in backyard swine populations included nasal and ocular discharge, coughing, sneezing, anorexia, lethargy, incoordination, paralysis of the hindquarters, rapid weight loss, pneumonia, and mortality [18–24]. The backyard swine without clinical signs of illness had a significantly higher IAV seroprevalence (2897/15693; 18.46%) than virological prevalence (69/9389; 0.73%). On the other hand, backyard swine with clinical signs of illness had a comparable serological (89/635; 14.01%) and virological (66/797; 8.28%) IAV prevalence. An overview of IAV sero- and virological prevalence in clinically healthy and sick backyard swine is provided in Fig. 4.

The highest number of studies that investigated IAV sero- and/or virological prevalence in backyard swine populations were reported from Asia (n = 11) followed by North America (n = 10), South America (n = 6), Africa (n = 6), and Europe (n = 1). We analyzed the sero- and virological prevalence of IAV in backyard swine in a continent-wise manner as presented below.

## Africa

### Benin and Cote d’ Ivoire

Following a previous outbreak of highly pathogenic avian H5N1 virus in West Africa, a study was conducted to determine the prevalence of other IAV subtypes in backyard swine populations of Benin and Cote d’ Ivoire during 2009–2010. In this surveillance, backyard farms were preferred for monitoring because the previous H5N1 outbreak, during 2006–2008, predominantly occurred in backyard flocks. Sixty-two and 1548 nasal swab samples were collected from backyard pigs in Benin and Cote d’ Ivoire, respectively, and tested using real-time RT-PCR; none of them were IAV positive, resulting in a negative molecular prevalence. In addition, 457 blood samples obtained from backyard pigs in Cote d’ Ivoire were tested with ELISA and hemagglutinin inhibition (HI) assays, which resulted in a negative IAV seroprevalence [25].

### Cameroon

The second study in Africa, which investigated IAV in backyard swine, was conducted in rural Cameroon during December 2009 and August 2012. Here, nasal swabs and sera samples were collected from 325 backyard swine. Only two swine were found infected with pandemic A(H1N1)pdm09 virus clade 1A.3.3.2 through real-time RT-PCR, while the HI assay detected pandemic A(H1N1)pdm09 virus clade 1A.3.3.2 and H3N2 virus antibodies in one swine serum [26], suggesting a low IAV sero- and virological prevalence.

### Kenya

A total of 1491 nasal swab samples were collected from backyard swine in Kibera, Nairobi, during 2010–2012. Real-time RT-PCR and virus isolation identified 11 (0.7%) pigs infected with pandemic A(H1N1)pdm09 virus clade 1A.3.3.2. The complete genomes of three of the pandemic A(H1N1)pdm09 virus clade 1A.3.3.2 viruses were sequenced in this study. Additionally, 13 (10.2%) out of 127 swine sera samples were found IAV positive using the ELISA assay, while the HI assay detected H1N1 and H3N2 virus-specific antibodies. The IAV specific antibodies were also detected in poultry raised simultaneously with swine. The HA genes of the isolated pandemic A(H1N1)pdm09 virus clade 1A.3.3.2 viruses were closely related to the human A(H1N1)pdm09 virus clade 1A.3.3.2 viruses reported from Kenya in 2009, which suggested human-to-swine transmission of the pandemic A(H1N1)pdm09 virus clade 1A.3.3.2 viruses in Kenya [27]. The second study in Kenya investigated IAV sero- and virological prevalence in backyard swine sera (n = 1990) and nasal swab (n = 2066) samples, respectively, during September 2013–April 2014. While the ELISA detected IAV-specific antibodies in 230 (11.56%) sera samples, suggesting past infection, none of the nasal swabs could amplify IAV specific sequences using RT-PCR, suggesting the absence of active IAV infection in these Kenyan backyard swine populations at the time of the surveillance [28].

### Uganda

There was only one study that investigated the serological prevalence of IAV in Ugandan backyard swine. This study reported that the IAV specific antibodies were detected in 26 (4.98%) of 522 clinically healthy swine sera samples which were collected from household swine in the Lira and Masaka districts in 2015 [29].

### Nigeria

**Fig. 5:**
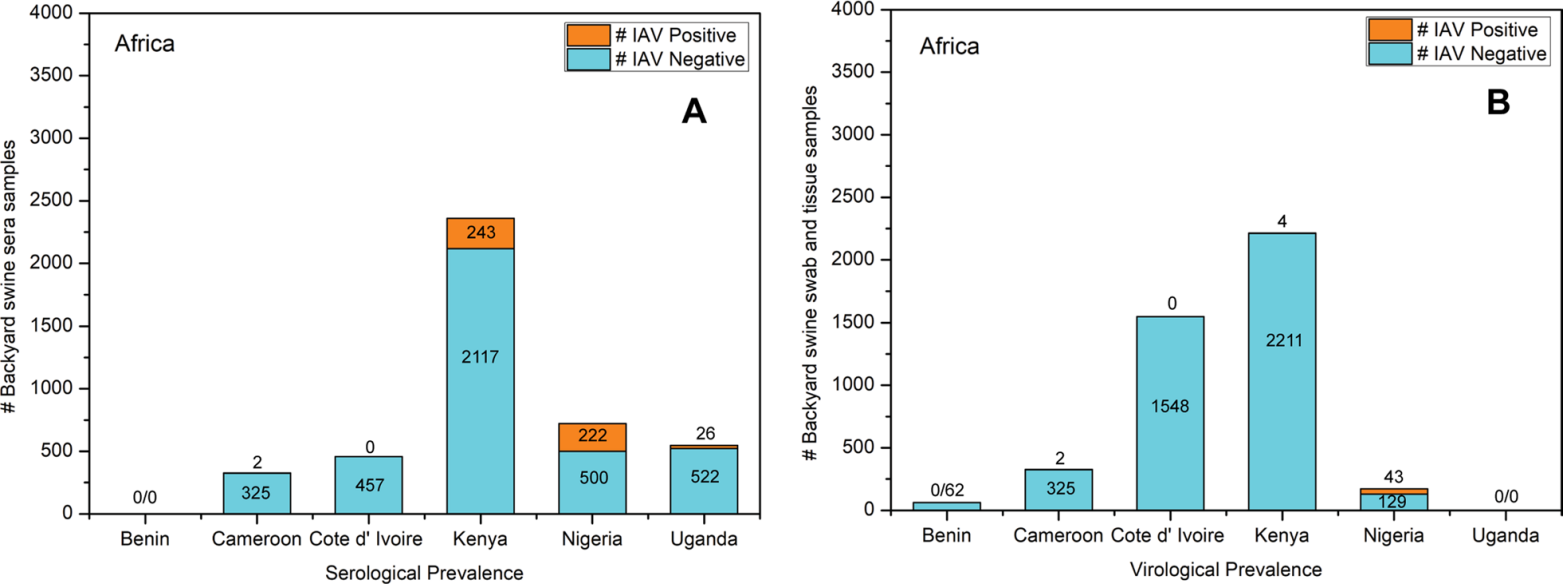
**a** Serological and **b** Virological prevalence of IAV in African backyard swine populations

Surveillance for IAV was conducted in Nigerian backyard swine during 2015–2016. Blood samples from 500 backyard pigs were collected for serological investigation, and 129 tracheal swabs and lung tissues were collected for virological investigation. The ELISA detected IAV-specific antibodies in 222 sera by HI assay, which confirmed pandemic A(H1N1)pdm09 virus clade 1A.3.3.2 (n = 14) and highly pathogenic H5N1 virus (n = 6) antibodies in sera samples. Real-time RT-PCR detected 43 IAV positive samples suggesting active IAV infections. Sanger sequencing confirmed the presence of highly pathogenic H5N1 viruses (n = 5) in Nigerian backyard swine [12]. Overall, while the IAV seroprevalence was detected in backyard swine in Cameroon, Kenya, Nigeria, and Uganda, active IAV infections (virological prevalence) were identified in Cameroon, Kenya, and Nigeria (Fig. 5).

### Asia

**Fig. 6:**
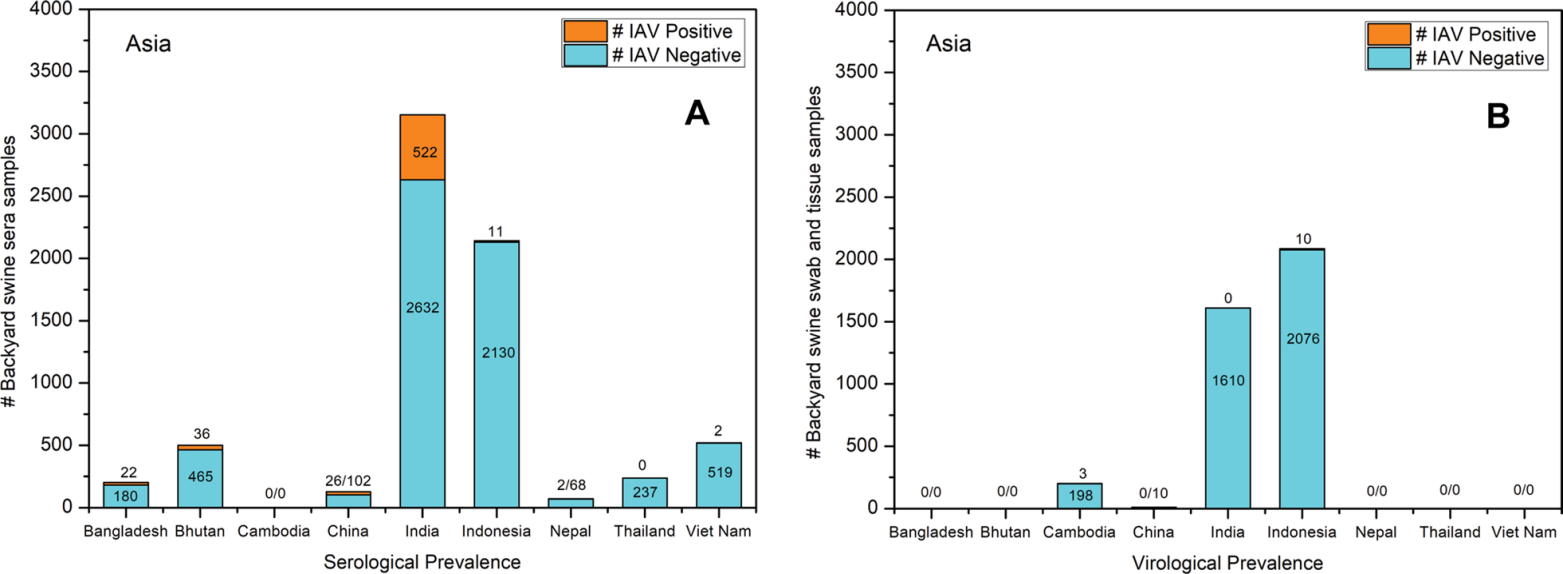
**a** Serological and **b** Virological prevalence of IAV in Asian backyard swine populations

A significantly low rate of past IAV infections (seroprevalence) and active infections (virological prevalence) were determined by various studies that were conducted in Asian countries, with a few exceptions, including China, India, Bangladesh, and Bhutan, where a relatively higher seroprevalence (25.49%, 19.83%, 12.22%, and 7.74%, respectively) was reported in the backyard swine. An overview of IAV sero- and virological prevalence in Asian backyard swine is represented in Fig. 6.

### Bangladesh

There was only one study that reported IAV sero-surveillance in backyard swine in Bangladesh. This study collected 180 sera samples from the backyard swine from July to December 2013. The ELISA assay detected IAV-specific antibodies in 22 (12.22%) swine sera [30] which suggested the circulation of IAV in backyard swine in Bangladesh in the past.

### Bhutan

A cross-sectional study during October 2011–February 2012 was conducted to identify the seroprevalence of IAV in rural backyard swine in Bhutan. Sera samples were collected from 465 backyard pigs in 69 villages and subjected to an ELISA test which detected IAV antibodies in 55 sera samples. All the ELISA positive sera were confirmed with the HI assay, which identified that 36 sera had pandemic A(H1N1)pdm09 virus clade 1A.3.3.2 antibodies while 35 sera had European swine H1N1 virus clade 1C.1 antibodies [31].

### Cambodia

Active IAV surveillance in rural Cambodia included 198 nasal swabs collected from backyard swine in 159 households located across 30 villages during May 2011–March 2013. Nasal swabs collected from a pig in 2011 and from two other pigs in 2012 were found IAV positive with real-time RT-PCR. The full-length genome sequencing of IAV positive swine identified H3N2 viruses in these three backyard swine [32].

### China

A sero-surveillance study detected H1N1 and H3N2 virus antibodies in 12 backyard swine sera samples collected during November 1993 in backyard farms in Nanchang, China. Households that reared swine and poultry in the backyards were selected for the study. Samples were also taken from the household members which determined the occurrence of H1N1 and H3N2 virus antibodies in human sera. Additionally, four H3N2 viruses were isolated from the human nasal swab samples. The data suggested transmission of H1N1 and H3N2 viruses from household members to the backyard swine [33]. Additionally, five H3N8, H4N4, H7N4, and H11N2 viruses were isolated from duck faeces [33], suggesting the circulation of various IAV subtypes in some of these backyard farms.

In another study, a European avian-like swine H1N1 virus clade 1C.1 was isolated from the tracheal specimen of a 3-years and 8-months old deceased boy at a family backyard farm who developed fever and dyspnea, which led to his admission to a local hospital where he later died due to further complications. Further investigation at the family backyard farm detected European avian-like swine H1N1 virus clade 1C.1 antibodies in two swine sera samples. Another family member was also found to be H1N1 positive while being asymptomatic. The swine and poultry were raised free-range in this backyard farm; however, poultry was found seronegative for IAV infections. Several of the pigs raised on this backyard farm were reported to have developed fatigue and had been slaughtered before the commencement of the study [23]. While the deceased boy was reported to not have had any known contacts with the backyard swine, which an elderly family member primarily raised, the data suggested a zoonotic transmission event from swine to the elderly family member who may have consequently transmitted the virus to the young boy in the family [23].

### India

Co-circulation of pandemic A(H1N1)pdm09 virus clade 1A.3.3.2 and seasonal human H1N1 influenza viruses were reported from Indian backyard swine during 2009- 2016. A total of 2632 sera and 1610 nasal swabs from backyard swine were either collected or received from ten Indian provinces. All the sera samples were analyzed for pandemic A(H1N1)pdm09 virus clade 1A.3.3.2 seroprevalence. While no virus could be isolated from the swine nasal swabs, 522 (19.83%) sera samples had antibodies for the pandemic A(H1N1)pdm09 virus clade 1A.3.3.2 suggesting a past exposure of these swine to the pandemic A(H1N1)pdm09 virus clade 1A.3.3.2. Additionally, 14.3% of the tested samples also had antibodies only for seasonal human H1N1 influenza virus, suggesting a past co-circulation of these viruses in Indian backyard swine populations [34].

### Indonesia

Two IAV surveillance studies were conducted in Indonesian backyard swine. The first surveillance collected 344 sera and 304 nasal and throat swabs from backyard swine in Indonesian villages during 2005. The microneutralization (MN) assay could not detect IAV antibodies in the sera samples. Similarly, all the 304 nasal and throat swabs were also negative for IAV with virus isolation and reverse-transcription-PCR [35], suggesting that the backyard swine populations under study were free from IAV. The second serological and virological surveillance took place in 2006 when 1786 sera and 1772 nasal swab samples were collected from backyard swine in Indonesian villages. The HI assay detected H5 antibodies in 11 swine sera samples, while the RT-PCR and Sanger sequencing identified ten H5N1 viruses in nasal swab samples [36]. This suggested previous as well as active H5N1 infection in Indonesian backyard swine.

### Nepal

A single sero-surveillance for IAV was conducted in the Nepalese backyard swine from August 2016–February 2017. Total 68 sera samples were collected from free-range backyard swine and tested for IAV antibodies using ELISA assay. Only two sera samples were found positive for IAV antibodies [37].

### Thailand

Only one sero-surveillance detected no IAV antibodies in 237 backyard swine sera samples collected from 74 households in Thailand during September 2016–February 2017 [38].

### Viet Nam

A serological study collected 519 sera samples from backyard swine in 158 villages located in Northern Viet Nam during April 2005–August 2006 for determining IAV seroprevalence. ELISA tests detected IAV antibodies only in two swine sera samples [39], suggesting a significantly low rate of past infection of these backyard swine.

### Europe

#### France

One highly pathogenic avian influenza H5N8 virus was detected in a backyard swine in France during an H5N8 outbreak among French poultry during 2016–2017. The HI assay confirmed that one backyard swine had antibodies against H5 clade 2.3.4.4b and identified that avian origin H5N8 virus would have been transmitted from domestic ducks raised on the backyard farm to the swine [40]. No clinical signs of disease were reported from the infected backyard swine. This was the only report of IAV in European backyard swine.

### North America

#### Costa Rica

In Costa Rica, nasal swabs were collected from 509 backyard swine from 25 observation units with clinical signs of influenza-like illness during October–November 2010. An observation unit was defined as a space that confined the pigs either within an entire backyard or the pigs physically confined within an individual barn. Real-time RT-PCR detected 11 (2.16%) pandemic A(H1N1)pdm09 virus clade 1A.3.3.2 positive nasal swab samples, which indicated a low rate of active IAV infection in these backyard swine [24].

#### Dominican Republic

Fifty-four sera samples collected from backyard swine in 36 premises in the Dominican Republic during August 2010 were tested for IAV seroprevalence using the HI assay, which detected H1N1 and H3N2 virus antibodies in 12 (22.2%) and 17 (31.5%) samples, respectively. These results suggested a past exposure of these backyard swine to the IAV. In this study, the human-to-swine transmission of IAV was suspected [22].

#### Guatemala

A study included collecting nasal swabs and sera samples from 426 backyard swine in 2010 and 2011 in Guatemala. Sera samples were tested with ELISA and HI assays, which identified 13 sera with A(H1N1)pdm09 virus clade 1A.3.3.2 and/or swine H1 and H3 virus antibodies. Interestingly, 52 nasal swabs were determined to be IAV positive, out of which four viruses were successfully isolated. While three isolates were confirmed to be pandemic A(H1N1)pdm09 virus clade 1A.3.3.2 viruses, one isolate was a human-like H3N2 virus. This investigation suggested a reverse-zoonotic transmission of human A(H1N1)pdm09 virus clade 1A.3.3.2 and H3N2 viruses to the backyard swine in Guatemala [19].

#### Haiti

Sera samples were collected from 109 backyard swine from 10 regions in Haiti during April 2010. The HI assay identified H1N1 virus antibodies in 24 (22.01%) and H3N2 virus antibodies in 13 (11.92%) sera samples [41] which suggested past infections of H1N1 and H3N2 viruses in backyard swine populations under investigation.

#### Mexico

Three studies have been conducted to detect IAV in Mexican backyard swine populations. The first study retrospectively analyzed 2094 backyard swine sera samples for the IAV antibodies from 2000 to 2009. The HI assay identified the highest seroprevalence of swine H1N1 virus (74%), which was followed by swine H3N2 virus (24.2%), pandemic A(H1N1)pdm09 virus clade 1A.3.3.2 virus (17.8%), and human H1N1 virus (1.3%) [42]. Intriguingly, the findings revealed the seroprevalence and, therefore, the past exposure of these Mexican backyard swine to the pandemic A(H1N1)pdm09 virus clade 1A.3.3.2 viruses. This was an interesting observation because it suggested that the pandemic A(H1N1)pdm09 virus clade 1A.3.3.2 viruses were circulating in the Mexican swine well before the emergence of the 2009 swine flu pandemic, which originated in Mexico. In the second study, nasal and rectal swabs from 23 backyard swine were collected from four backyard swine farms located in rural areas in Mexico during February–July 2016. Next-generation sequencing was conducted to study the virome of the backyard swine, which identified several RNA and DNA viruses in the samples under investigation; however, IAV was not detected in these samples [43]. The third study included nasal swabs (n = 175) of backyard swine, which were tested for IAV prevalence using a real-time RT-PCR assay. These samples were collected from a wetland which was located at a wild duck-backyard livestock interface in Mexico. The study's objective was to determine IAV circulation at this interface and identify and characterize the IAV subtypes. While none of the swine nasal swabs tested IAV positive, which ruled out active IAV infection in the backyard swine, three IAV subtypes, including H1N1, H3N2, and H5N2, were detected in the Mexican duck (*Anas diazi*), which emphasized the significance of active IAV surveillance in the region to monitor the possible future spillover [44].

#### Trinidad & Tobago (West Indies)

In a sero-surveillance conducted during October 2013–February 2015, 139 swine sera were collected from small backyard farms located on the island of Trinidad. In addition, 45 swine sera were collected from the small backyard farms located on the island of Tobago. While the ELISA assay detected IAV antibodies in 14 (10.07%) sera obtained from Trinidad, the sera from Tobago were negative for IAV seroprevalence. Among the ELISA positive samples from Trinidad, H3N2 and pandemic A(H1N1)pdm09 virus clade 1A.3.3.2 virus antibodies were detected by the HI assay [45]. These data suggested a past circulation of IAV, which appeared to be confined to the island of Trinidad, while no past IAV exposure was detected in the backyard swine on the island of Tobago in this investigation.

#### United States

Following a respiratory disease outbreak in the swine on a family farm in Wisconsin in October 1975, characterized by sneezing, coughing, and anorexia in the swine, an 8-year-old boy who had close contact with the swine in the household became ill with fever, headache, chills, abdominal pain, and sore throat. The swine H1N1 virus antibodies were detected in the serum sample of the boy 3 weeks after the onset of the illness. As a result, an investigation was initiated to ascertain the source of infection to the boy. Interestingly, the antibodies for the swine H1N1 virus were also detected in eight of the ten swine sera samples collected from the boy’s family farm [18]. These data suggested swine-to-human zoonotic transmission of the swine H1N1 virus on the Wisconsin family farm.

**Fig. 7:**
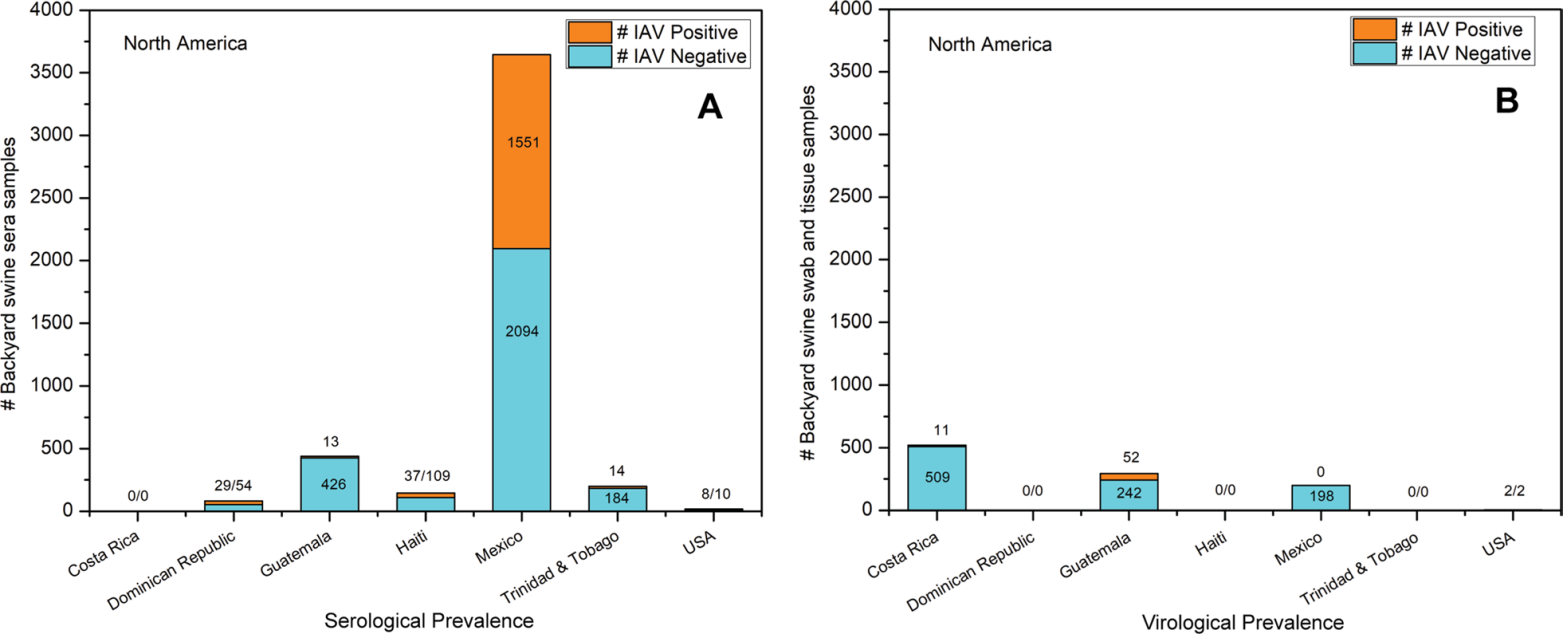
Serological (**a**) and virological (**b**) prevalence of IAV in North American backyard swine populations. The highest seroprevalence was reported in the Mexican backyard swine. The highest virological prevalence was identified in the backyard swine in Guatemala

In a more recent study, after observing the symptoms of pneumonia and rapid weight loss in one-month-old piglets at a small backyard piggery during November 2010 in Colorado, intestine, and lung tissue samples were submitted to the laboratory for diagnosis. Molecular diagnostics followed by virus isolation and genome sequencing for the hemagglutinin (HA) gene identified that the two piglets were infected with pandemic A(H1N1)pdm09 virus clade 1A.3.3.2 virus. Since the piggery was owned by a pharmacist who may have had occupational exposure to the A(H1N1)pdm09 virus clade 1A.3.3.2 virus, reverse-zoonotic transmission from the owner to the piglets was suspected [21]. An overview of IAV sero- and virological prevalence in North American backyard swine populations is represented in Fig. 7.

### South America

#### Brazil

An IAV serological surveillance during 2012 and 2014 consisted of 1667 backyard swine sera from 479 subsistence swine holdings in the Rio Grande do Sul province of Brazil. All the swine sera were initially screened for IAV antibodies using the ELISA assay. The ELISA positive sera (n = 111, 6.65%) were subtyped using the HI assay, which detected pandemic A(H1N1)pdm09 virus clade 1A.3.3.2 virus antibodies in 92 (5.51%) swine sera, while six sera had antibodies for H1N2 and five sera had antibodies for H3N2 viruses [46]. This was the only investigation from Brazil reporting IAV seroprevalence in backyard swine.

#### Chile

Chile reported the highest number of studies (n = 4) that conducted IAV surveillance in backyard swine populations. The first study investigating both IAV virological and serological prevalence was conducted in Central Chile during 2012–2014. A total of 67 nasal swabs and 127 sera were collected from backyard swine which were subjected to real time-PCR and ELISA tests, respectively. While all the nasal swabs were negative for IAV, only two sera had IAV antibodies suggesting a low circulation of IAV in backyard swine under investigation [47]. The second study reported active IAV infection in backyard swine during Spring 2013 and Fall 2014 and identified an H1N2 virus from a swine nasal swab sample [20]. Interestingly, poultry and geese were also identified as IAV positive at the same backyard farm, suggesting the risk of interspecies transmission of IAV. The third study identified an H1N2 virus from a nasal swab sample of a backyard swine in Central Chile [48]. This study included three nasal swabs and 266 sera of backyard swine collected during 2013–2015 in Central Chile. While the ELISA detected IAV antibodies in 86 (32.33%) swine sera samples, the HI assay detected IAV antibodies in only 22 (8.27%) sera. Subtypes identified were human H1N1, swine H1N1, swine H1N2, and pandemic A(H1N1)pdm09 virus clade 1A.3.3.2 viruses. Interestingly, 15 of the swine sera samples were positive for multiple IAV subtypes, as determined by the HI assay, suggesting the co-circulation of multiple IAV subtypes in backyard swine in Chile. In addition, active infection was detected in one of the nasal swabs which was identified as an H1N2 virus after sequencing [48]. The fourth surveillance study was conducted in backyard swine during September 2013 and July 2015. In this study, 64 sera and 39 nasal swab samples were collected. While four swine sera were IAV seropositive with ELISA, only one nasal swab was IAV positive using a real time-PCR assay [49]. None of the IAV positive samples in this study could be subtyped.

#### Peru

**Fig. 8:**
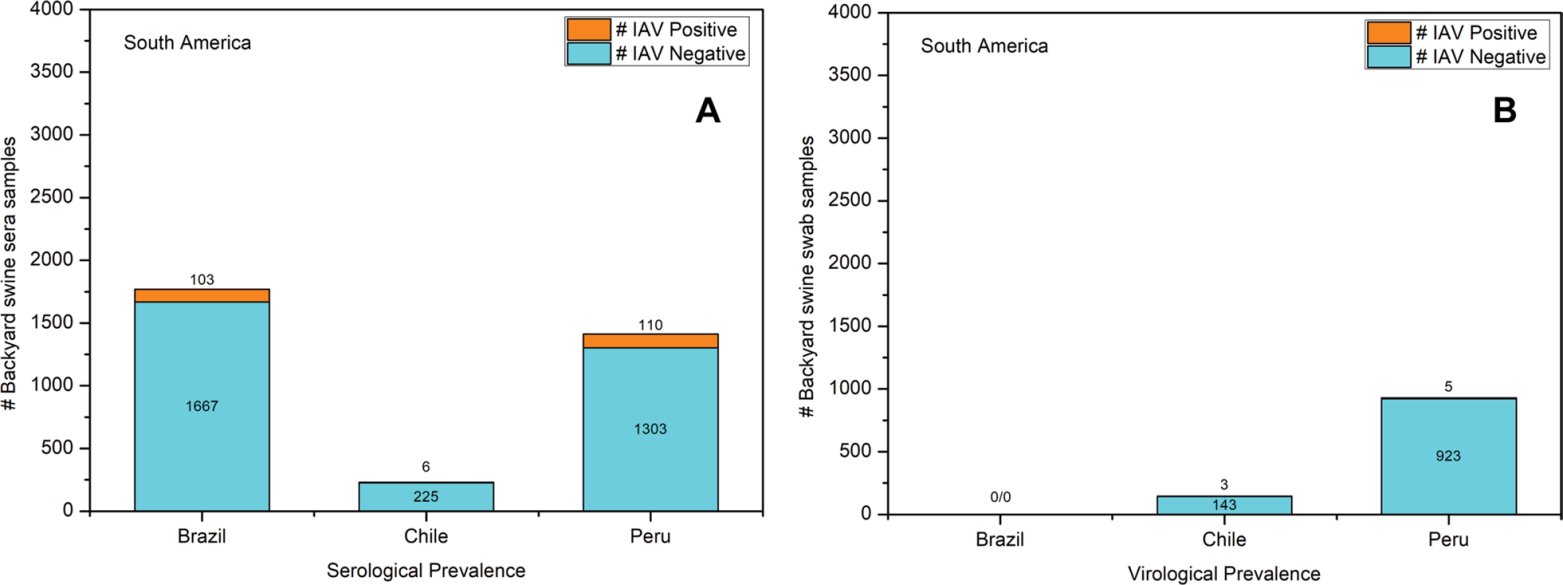
Serological (**a**) and virological (**b**) prevalence of IAV in South American backyard swine populations

A total of 1303 sera and 923 tracheal swab and lung tissue samples were collected at the time of slaughter from apparently healthy backyard swine in Tumbes, Peru, at four different times during March 2009–October 2011. While the HI assay detected pandemic A(H1N1)pdm09 virus clade 1A.3.3.2 virus antibodies in 110 (8.44%) sera, virus isolation and reverse transcription-PCR detected pandemic A(H1N1)pdm09 virus clade 1A.3.3.2 viruses in one lung tissue sample and four tracheal swabs only. Despite a significantly low molecular prevalence, the phylogenetic analysis determined more than one human-to-swine transmission events for these pandemic A(H1N1)pdm09 virus clade 1A.3.3.2 viruses in Tumbes [50]. An overview of IAV sero- and virological prevalence of South American backyard swine is represented in Fig. 8.

**Fig. 9:**
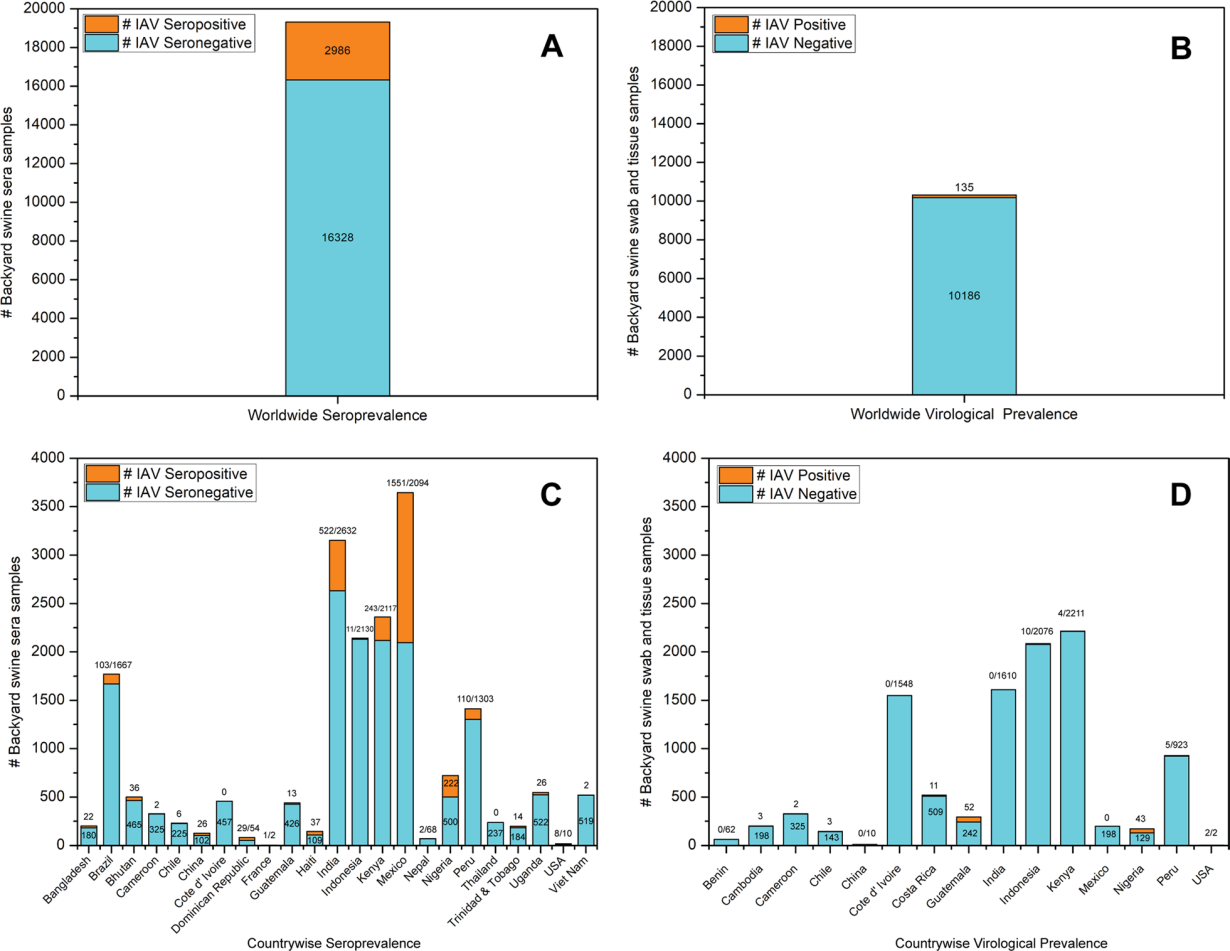
**a** Serological and **b** Virological prevalence of IAV in backyard swine populations globally **c** Serological and **d** Virological prevalence of IAV in backyard swine populations in various countries. It should be noted that the objectives of IAV surveillance may vary among countries; hence the data presented here may only be used as an indicator of IAV sero- and virological prevalence in the backyard swine populations

In summary, an overall significantly higher seroprevalence (18.28%; 2986/16328) (Fig. 9a) was detected in backyard swine populations compared to the virological prevalence (1.32%; 135/10186) (Fig. 9b). A relatively higher IAV seroprevalence was reported in backyard swine populations in Brazil, India, Kenya, Mexico, Nigeria, and Peru (Fig. 9c). More precisely, a higher seroprevalence of pandemic A(H1N1)pdm09 virus clade 1A.3.3.2 viruses were reported than other IAV subtypes in backyard swine in various countries. On the other hand, IAV active infections (virological prevalence) were reported from backyard swine in Cameroon, Kenya, Nigeria, Cambodia, Indonesia, Guatemala, Chile, Peru, and the USA (Fig. 9d). Notably, the IAV active infections included the subtypes of pandemic A(H1N1)pdm09 virus clade 1A.3.3.2, H1N2, H3N2, and H5N1 viruses in backyard swine populations.

### IAV transmission dynamics in backyard swine

**Fig. 10:**
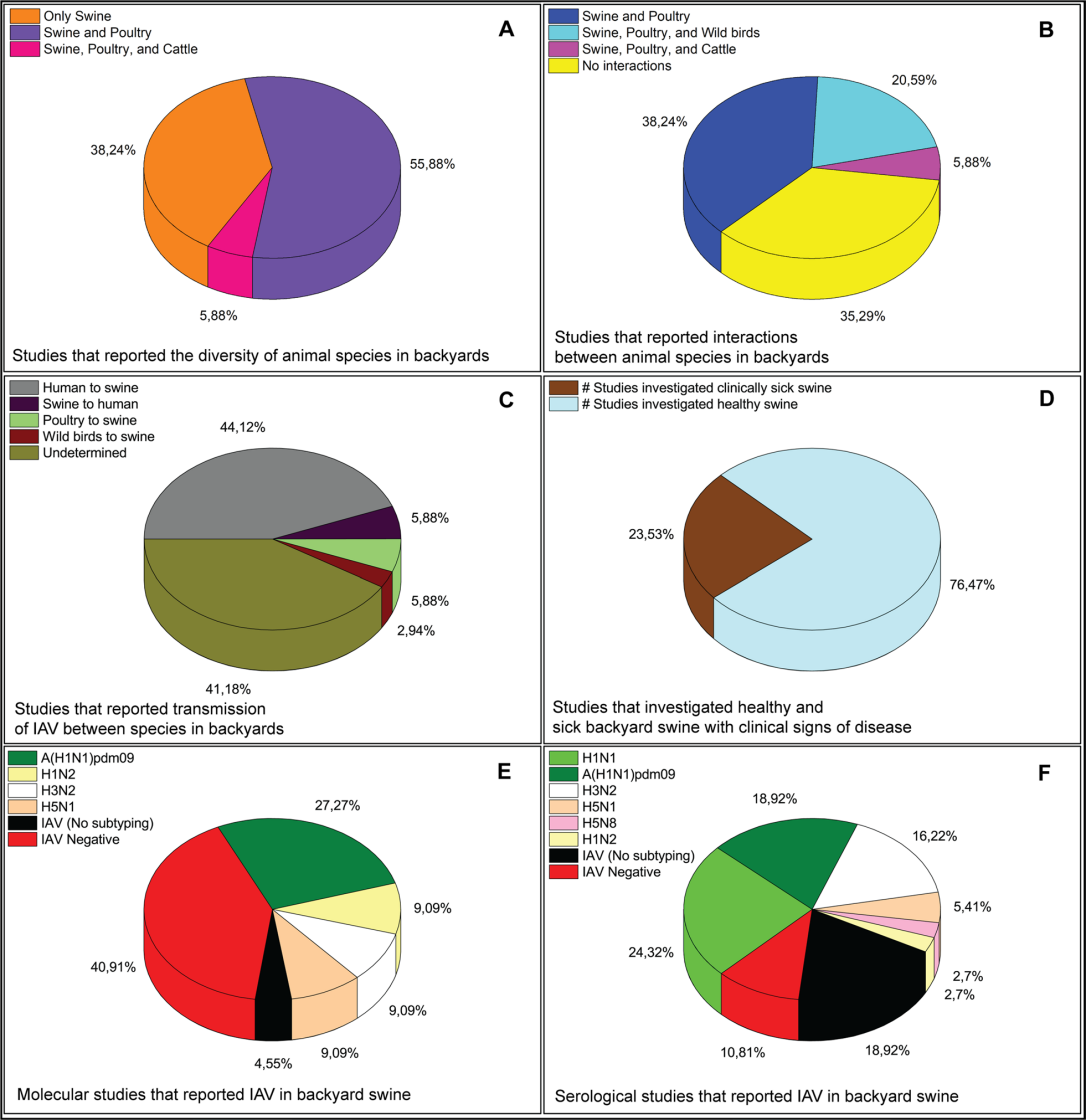
The global status of backyard swine farming along with transmission dynamics of IAV. **a** Several studies reported the presence of various animal species in swine backyard farms. **b** The number of studies that reported the interactions between backyard swine and other animal species in the backyards. **c** The number of studies that reported IAV interspecies transmission in the backyards. **d** The number of studies that investigated symptomatic and clinically healthy backyard swine. **e** Many molecular studies identified and characterized various IAV subtypes in backyard swine swabs and tissue samples. **f** The number of serological studies that identified and characterized different IAV subtypes in backyard swine sera samples.

While a few studies (n = 13; 38.24%) reported that swine were the only household animals in the backyards [19, 21, 22, 41, 43, 45], most of the studies reported either the presence of swine and poultry (n = 19; 55.88%) [20, 24, 44, 47–49] or swine, poultry, and cattle (n = 2; 5.88%) [42, 46] (Fig. 10a). Interestingly, 13 (38.24%) studies reported that swine and poultry regularly interacted in the backyards [12, 20, 23, 27, 28, 33, 35, 37, 39, 40, 47–49], while seven (20.58%) studies reported interactions among the wild birds, swine, and poultry [24–26, 30, 32, 36, 44]. Two (5.88%) studies reported interactions among the swine, poultry, and cattle [42, 46], while the other remaining studies (n = 12; 35.29%) did not mention any interactions between backyard swine and other animal species [18, 21, 22, 29, 31, 34, 38, 41, 45] (Fig. 10b). As far as the reports of interspecies IAV transmission were concerned, numerous studies suggested human-to-swine (reverse zoonotic) transmission of IAV (n = 15; 44.12%) [19, 21, 22, 24, 26, 27, 29, 31, 33, 34, 41, 42, 45, 46, 50] while swine-to-human (n = 2; 5.88%) [18, 23], poultry-to-swine (n = 2; 5.88%) [36, 40], and wild birds-to-swine (n = 1; 2.94%) [47] IAV transmissions within the backyards were also suggested. On the other hand, several studies (n = 14; 41.18%), including those that conducted sero-surveillance only [29–31, 37–41], could not determine the epidemiology of IAV transmission in the backyards (Fig. 10c). While only a few studies were aimed towards investigating IAV in swine having clinical signs of disease (n = 8; 23.53%) [18–24, 41], most of the studies included clinically healthy swine (n = 26; 76.47%) [12, 25–40, 42–50] for the detection of IAV (Fig. 10d).

The molecular investigations that utilized real-time RT-PCR and Sanger sequencing for detection and characterization of IAV reported that pandemic A(H1N1)pdm09 virus clade 1A.3.3.2 was the more frequently present IAV subtype (n = 6; 27.27%) [19, 21, 24, 26, 27, 50] in backyard swine populations compared to H1N2 (n = 2; 9.09%) [20, 48], H3N2 (n = 2; 9.09%) [19, 32], and H5N1 (n = 2; 9.09%) [12, 36] subtypes. Several molecular investigations (n = 9; 40.91%) could not detect IAV in backyard swine swab or tissue samples under investigation [23, 25, 28, 34, 35, 43, 44, 47], which resulted in a negative IAV prevalence, suggesting the absence of IAV active infection at the time of the investigation (Fig. 10e). A few serological investigations (n = 7; 18.92%) that detected IAV antibodies in backyard swine sera but did not report the subtype was because they only used ELISA assay [28–30, 37, 39, 47, 49] (Fig. 10f).

Serological studies that used HI assay reported the antibodies for H1N1 (n = 9; 24.32%), pandemic A(H1N1)pdm09 virus clade 1A.3.3.2 (n = 7; 18.92%), H3N2 (n = 6; 16.22%), H5N1 (n = 2; 5.41%), H5N8 (n = 1; 2.7%), and H1N2 (n = 1, 2.7%) viruses (Table 1).

**Table 1:** A summary of studies that reported IAV subtypes in backyard swine populations globally

Continent	Country	Serological methods	Antibodies detected	Citations	Molecular methods	IAV subtypes identified	Citations
Africa	Benin	None	None	[25]	Real time RT-PCR	None	[25]
	Cote d’Ivoire	ELISA, HI	None	[25]	Real time RT-PCR	None	[25]
	Cameroon	ELISA, HI	A(H1N1)pdm09 virus clade 1A.3.3.2	[26]	Real time RT-PCR, Sanger sequencing	A(H1N1)pdm09 virus clade 1A.3.3.2	[26]
	Kenya	ELISA, HI	IAV, H1N1, H3N2	[27, 28]	Real time RT-PCR, virus isolation	A(H1N1)pdm09 virus clade 1A.3.3.2	[27]
	Nigeria	ELISA, HI	A(H1N1)pdm09 virus clade 1A.3.3.2, H5N1	[12]	Reverse transcription-PCR, real time RT-PCR, Sanger sequencing	H5N1	[12]
	Uganda	ELISA	IAV	[29]	None	None	
Asia	Bangladesh	ELISA	IAV	[30]	None	None	
	Bhutan	ELISA, HI	A(H1N1)pdm09 virus clade 1A.3.3.2, swine H1N1 clade 1C.1	[31]	None	None	
	Cambodia	None	None		Real time RT-PCR, Sanger sequencing	H3N2	[32]
	China	HI, NI	H1N1, H3N2	[23, 33]	Real time RT-PCR, virus isolation	None	[23]
	India	ELISA, HI	H1N1, A(H1N1)pdm09 virus clade 1A.3.3.2	[34]	Virus isolation	None	[34]
	Indonesia	HI, MN	H5N1	[35, 36]	Virus isolation, reverse transcription-PCR, Sanger sequencing	H5N1	[35, 36]
	Nepal	ELISA	IAV	[37]	None	None	
	Thailand	ELISA	None	[38]	None	None	
	Viet Nam	ELISA	IAV	[39]	None	None	
Europe	France	ELISA, HI	H5N8	[40]	None	None	
North America	Costa Rica	None	None		Real time RT-PCR, virus isolation, Sanger sequencing	A(H1N1)pdm09 virus clade 1A.3.3.2	[24]
	Dominican Republic	HI	H1N1, H3N2	[22]	None	None	
	Guatemala	ELISA, HI	A(H1N1)pdm09 virus clade 1A.3.3.2, H3N2	[19]	Real time RT-PCR, virus isolation, Sanger sequencing	A(H1N1)pdm09 virus clade 1A.3.3.2, H3N2	[19]
	Haiti	ELISA	H1N1, H3N2	[41]	None	None	
	Mexico	HI	Human H1N1, Swine H1N1, Swine H3N2, A(H1N1)pdm09 virus clade 1A.3.3.2	[42]	Real time RT-PCR, MiSeq	None	[43]
	Trinidad & Tobago	ELISA, HI	A(H1N1)pdm09 virus clade 1A.3.3.2, H3N2	[45]	None	None	
	USA	HI	Swine H1N1	[18]	Real time RT-PCR, virus isolation, Sanger sequencing	A(H1N1)pdm09 virus clade 1A.3.3.2	[21]
South America	Brazil	ELISA, HI	H1N2, H3N2, A(H1N1)pdm09 virus clade 1A.3.3.2	[46]	None	None	
	Chile	ELISA	IAV	[47, 49]	Real time RT-PCR, virus isolation, Sanger sequencing	H1N2	[20, 48, 49]
	Peru	HI	A(H1N1)pdm09 virus clade 1A.3.3.2	[50]	Reverse transcription-PCR, Sanger sequencing	A(H1N1)pdm09 virus clade 1A.3.3.2	[50]

## Discussion

Though numerous reports were available on IAV prevalence in organized (commercial) swine farms globally during the twentieth century [51], the IAV surveillance in rural backyard swine populations remained neglected until the emergence of the swine flu pandemic in 2009 in Mexican swine [52]. The swine flu pandemic of 2009 appears to have acted as a catalyst for IAV surveillance in backyard swine populations because most of the studies (85.29%; 29/34) were commenced after the swine flu pandemic hit in March 2009. Within a relatively short period during 2009–2021, total 34 studies have been reported providing valuable insights on IAV transmission dynamics in backyard swine populations in various countries [19, 38, 49]. While the human-origin pandemic A(H1N1)pdm09 virus clade 1A.3.3.2 viruses were more commonly reported in backyard swine, the human H1N1 and H3N2 viruses were also detected in backyard swine in various countries suggesting reverse-zoonotic transmission events from human-to-swine. Intriguingly, the presence of avian-origin highly pathogenic viruses including H5N1 [12] and H5N8 [40] suggest the possibilities of avian-to-swine transmission. In particular, the presence of highly pathogenic avian H5N1 viruses in Nigerian backyard swine is of interest because Nigeria falls within the East Africa–West Asia flyway of the migratory birds [53] used by long-distance migratory wild birds for intercontinental migration for overwintering [54]. The occurrence of significantly large numbers of highly pathogenic H5N1 viruses in poultry populations in Nigeria [12, 15], might be attributed to the migratory wild birds in the country. Since these birds can potentially introduce exotic IAV subtypes into the domestic bird populations, the interactions of swine, poultry, and wild birds in backyard farms pose a high risk of IAV disease transmission.

Similarly, South Africa, due to its unique geographic location, is a favourable destination for wild birds for over-wintering, and falls within the East Africa–West Asia flyway and East Atlantic flyway of wild migratory birds [53]. In recent years, numerous avian influenza virus subtypes have been reported in wild and domestic avian species in South Africa which poses a significant threat in terms of avian to swine spillover of these viruses [11, 55–60]. We recently reported avian (chicken, duck, pigeon, mallard, and other wild birds) to swine spillover and adaptation of eleven IAV subtypes in swine populations globally [61] which suggested that several of these viruses have been already adapted in swine. The adaptation of IAV in swine poses a threat regarding further spillover and disease outbreaks and threatens public health. A recent study from Chile suggested that the interaction of backyard swine with domestic poultry or wild birds may facilitate the transmission of IAV within the backyards [49].

While IAV active infection was detected only in a few backyard swine in Asia (Cambodia and Indonesia) [32, 36], seroprevalence was reported from Bangladesh [30], Bhutan [31], China [23, 33], India [34], Indonesia [36], Nepal [37], and Viet Nam [39]. We believe this was due to the surveillance objectives, which varied between countries. For example, six studies attempted only to detect seroprevalence of IAV in backyard swine in Asia [30, 31, 33, 37–39], while four investigated both sero- and virological prevalence [23, 34–36] and remaining one study investigated only virological prevalence in the Asian backyard swine [32]. Of note was the occurrence of only two surveillance studies in Chinese backyard swine [23, 33] despite China being the largest swine producer globally [62, 63]. Most importantly, it has been considered an epicentre of IAV disease [64, 65].

In contrast, numerous studies have reported several IAV subtypes in Chinese commercial swine populations [51]. The limited investigation in Chinese backyard swine suggests that emphasis in China has been placed on large-scale commercial swine farms, which are the major suppliers of pork meat to the Chinese consumers. The neglect of IAV disease surveillance on backyard swine farms in China could have far-reaching consequences given the region's widespread circulation of avian and human origin IAV subtypes [51]. For example, a recent study from China documented a novel subtype (G4 EA H1N1), which is a reassortant avian-like H1N1 swine IAV subtype of clade 1C.2.3 with genes from pandemic A(H1N1)pdm09 virus clade 1A.3.3.2 at multiple Chinese commercial swine farms with evidence of zoonotic transmission to the swine farm workers [66]. While many studies have reported virological and serological prevalence of IAV on commercial large-scale swine farms in China [51, 67], limited information of IAV disease in backyard swine populations in China reflects the negligence regarding IAV surveillance in backyard swine farms in China. Similarly, the limited number of sero- and/or virological surveillance studies in other Asian countries also reflects the neglect of IAV disease surveillance in rural Asian settings having backyard swine compared to commercial swine populations [51]. The true disease status and evolution of IAV in backyard animals, including swine, cannot be determined in the absence of active IAV surveillance.

Most intriguingly, the seroprevalence of pandemic A(H1N1)pdm09 virus clade 1A.3.3.2 virus in backyard swine populations in Mexico during 2000–2009 suggested the occurrence and circulation of pandemic A(H1N1)pdm09 virus clade 1A.3.3.2 viruses in Mexican backyard swine well before the emergence of the 2009 swine flu pandemic in Mexico [42]. This suggested that the continued circulation of pandemic A(H1N1)pdm09 virus clade 1A.3.3.2 virus in Mexican swine resulted in the acquisition of mutations for efficient mammalian transmission, and thus triggered the pandemic. The sero- and virological prevalence of human-origin H1N1, H3N2, and pandemic A(H1N1)pdm09 virus clade 1A.3.3.2 viruses in North American [21, 45] and H1N2, H3N2, and pandemic A(H1N1)pdm09 virus clade 1A.3.3.2 viruses in South American [48, 50] backyard swine populations indicated multiple human-to-swine transmission events. These data reiterated that human–swine interactions within the households or backyards may trigger zoonotic and reverse-zoonotic transmission of IAV endangering public health.

It has been established that the IAV infected swine may start virus shedding one day post-infection (dpi) while the virus can be shed up to 5 dpi [68] and hence can be detected successfully in swine nasal swab samples up to 5 dpi [69]. Additionally, swine's onset of disease symptoms occurs 1–3 dpi, while recovery starts about a week post-infection [70]. These data suggested that a delay in obtaining the nasal swabs from a clinically sick swine might result in a negative molecular detection. Interestingly, it should also be noted that other virus pathogens, including Porcine astrovirus type-4 (PAstV-4) can also cause acute respiratory illness in swine mimicking the symptoms related to influenza-like illness [71]. However, most of the studies investigated IAV in clinically healthy backyard swine which had no clinical signs of influenza disease, it appears to be one of the reasons why a low rate of active IAV infection was detected in clinically healthy backyard swine under investigation (69/9389; 0.73%). On the other hand, the HI assay can effectively detect IAV antibodies in swine sera at seven dpi while the peak may reach up to two-to-three weeks post-infection [72, 73] therefore the HI assay would be able to detect the IAV antibodies in clinically symptomatic swine, which appears to be the case in backyard swine sero-surveillances where a comparable percentage of serological IAV prevalence was detected in clinically healthy (2897/15693; 18.46%) and clinically symptomatic backyard swine (89/635; 14.01%).

It is evident that widespread prevalence and circulation of various avian influenza viruses in wild birds and domestic poultry poses a constant threat to backyard swine farming, given the inadequate biosecurity measures, and therefore needs continuous monitoring of IAV disease. Therefore, adequate biosecurity on the backyard swine farms is recommended, with minimized direct human–swine interactions to reduce the possibility of zoonotic and reverse-zoonotic IAV transmission, thereby safeguarding public health. These findings reiterate the need for ongoing surveillance to track IAV circulation and evolution in backyard swine populations.

## Conclusions

Backyard swine farms rearing both swine and poultry remain at a high risk of IAV interspecies transmission from domestic poultry to swine. In addition, migratory wild birds pose a significant threat and may introduce exotic IAV subtypes to the backyard swine. The possibility of zoonotic and reverse-zoonotic transmission between swine and humans also persists within the backyard farms. The occurrence of pandemic A(H1N1)pdm09 virus clade 1A.3.3.2, highly pathogenic avian H5N1, and several other IAV subtypes in backyard swine populations should be of concern as this may cause disease outbreaks in swine as well as in the exposed human populations. The occurrence of both human and avian IAV subtypes in backyard swine may facilitate their evolution, representing public health risk. A policy of active IAV surveillance in backyard swine populations should be implemented to track their molecular evolution.

## Data Availability

Not applicable.

## Abbreviations

Dpi: Days post-infection
ELISA: Enzyme-linked immunosorbent assay
IAV: Influenza A virus
HI: Hemagglutinin inhibition
MN: Microneutralization
PRISMA: Preferred reporting items for systematic reviews and meta-analysis
RT-PCR: Reverse-transcription-polymerase chain reaction
